# Supplementary material for: Green Space Exposure and Obesity in the Mexican Adult Population
Source: Int J Environ Res Public Health. 2022 Nov 16;19(22):15072. doi: 10.3390/ijerph192215072 (PMC9690096; doi:10.3390/ijerph192215072)
Supplement: Supplementary file 1 [file ijerph-19-15072-s001.zip › ijerph-1989936-supplementary.pdf]

Table S1. Characteristics socio-demographic of Mexican adults' population aged 20 to 59 participants in ENSANUT 2018-19, by quartiles of NDVI (buffer 1230m).

**Characteristic**	**TOTAL**	**NDVI (buffer 270m)**			
n= 12,631* ***N= 59,710,238)****		**Quartile 1**	**Quartile 2**	**Quartile 3**	**Quartile 4**
NDVI					
Buffer 1230m (mean ± SD)	0.29 ± 0.0031	0.11 ± 0.0020	0.21 ± 0.0033	0.32 ± 0.0038	0.48 ± 0.0065
Sex					
Male (%)	42.2	42.11	42.71	41.75	42.15
Female (%)	57.8	57.89	57.29	58.25	57.85
Age (years) (mean ± SD)	38.22 ± 0.19	39.14 ± 0.35	38.46 ± 0.40	37.41 ± 0.29	37.11 ± 0.38
BMI (mean ± SD)*	28.87 ± 0.08	29.25 ± 0.17	28.73 ± 0.15	28.97 ± 0.16	28.29 ± 0.14
Normal weight (BMI <25 kg/m^2) (%)	24.56	23.71	25.05	23.09	26.93
Obesity (BMI ≥25 kg/m^2) (%)	75.44	76.29	74.95	76.91	73.07
Education level (%)					
< High School	53.51	44.88	49.2	58.03	69.55
High School Certificate	26.86	29.16	30.3	24.71	20.68
> High School, Bachelor's degree or higher	19.63	25.96	20.5	17.26	9.76
Socioeconomic status (%)					
1st tertile (*low*)	28.38	11.85	20.43	36.13	59.62
2nd tertile (*medium*)	33.57	33.6	36.03	36.38	27.65
3rd tertile (*high*)	38.04	54.56	43.54	27.49	12.73
Region (%)					
Northeast	20.21	43.08	16.12	3.68	1.61
Central	33.01	21.43	38.53	48.52	30.99
Mexico City	16.41	30.06	16.81	7.01	1.31
South	30.37	5.43	28.54	40.78	66.08
Area (%)					
Urban	78.95	97.59	89.41	72.56	39.54
Rural	21.05	2.41	10.59	27.44	60.46
Physical activity moderate and vigorous, minutes/week** (%) ***[n= 10, 188 N=47,451,492]****					
Not Active <150 minutes	18.79	20.01	20.44	17.18	16.07
Active >150 minutes	81.21	79.99	79.56	82.82	83.93

Abbreviations: NDVI= Normalized Difference Vegetation Index; BMI= Body Mass Index; SD= Standard Deviation. * WHO classification. ** WHO guidelines on physical activity and sedentary behavior. *** *n*= sample size N= expanded sample. *All p-value*<0.05
